# ‘Comment on Saumitou et al. (2017): Elucidation of the genetic architecture of self‐incompatibility in olive: evolutionary consequences and perspectives for orchard management’

**DOI:** 10.1111/eva.12494

**Published:** 2017-06-29

**Authors:** Catherine Breton, Georgios Koubouris, Pierre Villemur, André Jean Bervillé

**Affiliations:** ^1^ Institut des Sciences de l’Évolution de Montpellier (ISE‐M) UMR CNRS 5554 Montpellier Cedex 5 France; ^2^ Institute for Olive Tree, Subtropical Crops & Viticulture Hellenic Agricultural Organization ‘Demeter’ (ex. NAGREF) Chania Greece; ^3^ Montpellier SupAgro Montpellier Cedex France; ^4^ INRA UMR‐DIAPC 1097 Montpellier Cedex 1 France

**Keywords:** andromonoecious species, dominance, *europaea* var. *europaea*, genetic control, *Olea europaea* subsp., pollinizer, sporophytic plant mating system

## Abstract

The new self‐incompatibility system (SI) was presented by Saumitou‐Laprade, Vernet, Vekemans et al. (2017). *Evolutionary Applications* based on 89 crosses between varieties in the olive tree. Four main points are not clear. We are examining here as follows: (i) the assertion that the self‐incompatibility system is sporophytic was not sustained by pollen germination data; (ii) surprisingly, the new model does not explain that about one‐third of pairwise combinations of olive varieties leads to asymmetric fruit setting; (iii) DNA preparation from one seed may contain two embryos, and thus, embryos should be separated before seed extraction; (iv) although effective self‐fertility in olive varieties was reported by many studies, the DSI model fails to explain self‐fertility in some olive varieties. Moreover, we cannot discuss result data, as science cannot be verified because variety names were encoded, this does not allow comparison of data with previous works. The DSI model on olive self‐incompatibility should explain more features than the model based on four dominance levels shared by six S‐alleles. Perspectives for orchard management based on this model may face serious limitations. An olive variety does not have a fifty percent chance of cross‐incompatibility, but surely fewer, and thus, the sporophytic system limits fruit production. Evolutionary perspectives of self‐incompatibility in Oleaceae should include data from the Jasmineae tribe that displays heterostyly SI.

## 1 INTRODUCTION

Saumitou‐Laprade et al. (2017) presented a new self‐incompatibility system (SI) based on 89 crosses between varieties in the olive tree. The authors have indicated that there are doubts on the sporophytic SI (SSI) in the olive, published by Breton and Bervillé (2012). Predictions from the SSI model have always matched experimental data based on fruit setting (Breton et al., 2014; Farinelli, Breton, Famiani, & Bervillé, 2015); moreover, they showed the scale of dominance shared between 6 S‐alleles. The Breton et al.'s model was sustained by all cross data and some diagnostics for SI based on pollen germination (Bradley & Griggs, 1963; Ouksili, 1983), and so far, in more than three thousand crosses (see references in Farinelli et al., 2015; Koubouris, Breton, Metzidakis, & Vasilakakis, 2014). No ambiguity has appeared to identify the sporophytic system, because all these authors displayed reciprocal crosses with opposite fruit sets (Gerstel, 1950). Gerstel (1950) based his studies on Guayule (*Parthenium argentatum* Gray).

It is unclear to us how Saumitou‐Laprade et al. (2017) could conclude on SSI after pollen germination tests and a few controlled crosses under pollination bags although they only observed symmetric compatibility or incompatibility for each pairwise combination of olive varieties. They observed 1:1 segregation for self‐fertility in pseudo‐backcross progenies Oit64xOit27 (which denomination is Oit64?). We have to believe because the cross remains unidentified. Such a genetic structure progenies is not common in genetic analysis. Some of the authors in Saumitou‐Laprade et al. (2017) also handle the offspring Picholine marocaine x Picholine in which the 1:1 segregation of self‐fertility should be checked. Breton, Farinelli, Koubouris, and Bervillé (2016) have shown than the self‐fertility level depends on the S‐allele pair and on modifiers which co‐segregated with the S‐loci.

Also Saumitou‐Laprade et al. (2017) provided the Collani et al. (2012) reference to sustain SSI. Until now, a SSI system resulted from cross results data, based on fruit set or on pollen germination tests. It is very rare that sporophytic SI is verified through molecular data, except in Brassiceae (Chookajorn, Kachroo, Ripol, Clark, & Nasrallah, 2004). Thus, it remains to be given, which crosses between identified varieties show in Saumitou‐Laprade et al. (2017) that SI is sporophytic yet?

Saumitou‐Laprade et al. (2017) introduced 2 S‐alleles (S1 and S2) and displayed symmetric diagnostics for compatibility in G1 and G2 groups (Table 1). Symmetry in compatibility or in incompatibility for pairwise combinations of olive varieties is observed and verified based on fruit setting in about half of pairwise combinations of olive varieties (Table 3 in Breton et al., 2014). Furthermore, several authors have reported that for the (more or less) other half of olive crosses, pairwise combinations of olive varieties show asymmetric fruit set (Musho, 1977; Ouksili, 1983; Villemur, Musho, Delmas, Maamar, & Ouksili, 1984; Moutier, Terrien, Pécout, Hostalnou, & Margier, 2006; Farinelli et al. 2010; Spinardi & Bassi, 2012; Farinelli et al., 2015). The proportion is more or less 50% depending on the set of varieties sampled for the study by each team.

**Table 1:** Asymmetry and symmetry for fruit setting in pairwise combinations of varieties in the two directions of crosses

Host variety	S‐allele pair	Pollen donor	Pollen S‐determinant	Cross Succ^a^/Fail^a^	Because of S‐allele	Host self‐fertility	Symmetry/Asymmetry
Picholine	*R1R3*	Manzanilla	R2	Succ	*R2*	None	Asymmetry
Manzanilla	*R1R2*	Picholine	R1R3	Fail	*R1*	None	
Picholine	*R1R3*	Tanche	R2	Succ	*R2*	None	Asymmetry
Tanche	*R2R3*	Picholine	R1R3	Fail	*R3*	Little	
Amellau	*R3R4*	Tanche	R2	Succ	*R2*	None	Asymmetry
Tanche	*R2R3*	Amellau	R3	Fail	*R3*	Little	
Grossane	*R1R5*	Aglandau	R2	Succ	*R5*	Little	Asymmetry
Aglandau	*R2R5*	Grossane	R1R5	Fail	*R5*	Little	
Manzanilla	*R1R2*	Belgentier	R6	Succ	*R2*	None	Asymmetry
Belgentier	*R2R6*	Manzanilla	R2	Fail	*R2*	Little	
Frantoio	*R4R5*	Leccino	R1R5	Fail	*R5*	High	Symmetry
Leccino	*R1R5*	Frantoio	R5	Fail	*R5*	Little	

The S‐allele pair in column ‘S‐allele pair’ infers that both determinants are present in stigma and style, encoded [RxRy]. Host self‐fertility: none = 0, Little = 0 <  to 0.2, high above = 0.2 fruit/100 hermaphroditic flowers; Succ: success. Fail: failure.

a Success and failure means that fruit numbers is, respectively, above and below thresholds. Manzanilla is from Spain, Frantoio, and Leccino are from Italy. All other varieties are from France; asymmetry means that fruit set in reciprocal crosses are opposite; symmetry means that fruit setting in both directions of reciprocal crosses either both failed or both succeeded.

The explanation for asymmetry of fruit setting has been given in Breton et al. (2014) and asymmetry in fruit setting proved the sporophytic model as pointed out by Gerstel (1950). In one direction, the cross leads to fruit set because of compatibility, and in the other direction, the cross fails because of incompatibility. However, in some peculiar situations, asymmetry may occur because of male sterility of one olive variety (Besnard, Khadari, Villemur, & Bervillé, 2000; Villemur et al., 1984), and because one variety is self‐fertile (Table 1) (Breton et al., 2016; Spinardi & Bassi, 2012).

Such pairs of asymmetric crosses are due to the differential S‐allele expression in the female and the male parts (see references in Saumitou‐Laprade et al., 2017). This has been shown in crosses for several species harboring a SSI system in guayule, sunflower, and hazelnut (Gerstel, 1950; Mehlenbacher, 1997; Ségala, Ségala, & Piquemal, 1980).

The DSI model does not explain the asymmetry reported in other studies based on crossing experiments and described in half of pairwise combinations. Shortly, the authors have, in no way, refuted the model proposed by Breton et al. (2014) based on six S‐alleles, which takes into account that the dominance scale (four levels) *R6 *>* R2 *>* R1 *=* R3 *=* R5 *>* R4* introduces asymmetry for fruit setting in some pairs of crosses. Moreover, encoding olive varieties, as ‘Oitxy, CBNMedxy, and OWGBxy, for Italy, France, and Morocco, respectively’, does not allow comparison of the results by Saumitou‐Laprade et al. (2017) with previous studies, except for the pair Leccino–Dolce Agogia, respectively, Oit27 and Oit15.

In the olive tree, such pairs of ♀host tree x ♂pollen donor have been reported and detailed, see Breton et al. (2014): in the example ♀Picholine x ♂Manzanilla, the cross succeeded, whereas ♀Manzanilla x ♂Picholine failed (very poor or no fruit set). Failure was explained for the first time by coding the S‐alleles *R1* to *Rn*; here, the *R1* S‐allele encoding the R1 determinant (encoded [R1] in the style) was supposed to be present both in Picholine (*R1R3*) and Manzanilla (*R1R2*), but the pollen of Manzanilla does not carry the R1 S‐determinant, because of dominance of *R2* to *R1* (*R2 *>* R1*), and thus, Manzanilla pollen is coded R2 as Mehlenbacher (1997) suggested to do in hazelnut.

Recently, Breton et al. (2016) have shown a correlation between the *S*‐allele pair and the level of self‐fertility. In some pairwise combinations of varieties, when the host variety is self‐fertile enough, fruit setting occurs under the bag and the final diagnostic for cross compatibility could be erroneous. Indeed, compatibility or incompatibility diagnostic has not been given to the pair Frantoio and Leccino (Spinardi & Bassi, 2012), because the origin of fruit remains inexplicable. Consequently, a column was added in Table 1 of the present study to show whether fruit setting in the host may be attributed to self‐pollination and not to foreign pollen. Controls by paternity tests have not been performed at this time. Thus, some examples of pairs of varieties that lead to asymmetric crosses are given in Table 1, with reference to Breton et al. (2014) and Farinelli et al. (2015), based on the list of varieties (#105) deciphered for the S‐allele pair, so far (C. M. Breton, D. Farinelli, G. Koubouris, F. Famiani, A. J. Bervillé, Unpublished). (Table 1).

A model is validated when its predictions match with tested out data. In the Breton et al.'s model, all predictions for cross successes and failures have been satisfied without any exceptions. In Saumitou‐Laprade et al. (2017), we cannot find any prediction based on the model, which would have been experimented in orchards or in controlled crosses.

Saumitou‐Laprade et al. (2017) have shown the number of fruit obtained under bags in Table 4. Comparison between olive varieties requests standardization of fruit setting, because between varieties, inflorescences do not carry the same number of flowers (between 10 and 60), and moreover, between varieties, the proportion of hermaphroditic flowers varies considerably (9% in Lucques – 100% in Frantoio and Salonenque). The olive tree is given as an andromonoecious species. Thus, the number of hermaphroditic flowers has to be counted before introduction of the foreign pollen, enabling to standardize fruit numbers per 100 hermaphroditic flowers to compare varieties (Farinelli et al., 2015). This has not been given in materials and method section by Saumitou‐Laprade et al. (2017), and thus, fruit numbers refer to an unknown number of hermaphroditic flowers. Bradley and Griggs (1963) have underlined that fruit should be counted no later than 8 weeks after pollination; otherwise, their number may be affected by other parameters than SI. Saumitou‐Laprade et al. (2017) have counted fruit at maturity, 6 months after fertilization has occurred.

Furthermore, DNA preparation from fruit does not follow the Díaz’ method, because Díaz, Martín, Rallo, and De la Rosa (2007) have obtained DNA, not from embryos, but from leaves of seedlings after germination of embryos. Paternity tests are therefore doubtful here: as one olive seed may contain up to two embryos from different fathers – if crosses have occurred (Farinelli, Pierantozzi, & Palese, 2012; Marchese et al., 2016). Obviously, Marchese et al. (2016) have prepared DNA from embryos, and thus DNA profiles show some mismatching to the correct father profile. Thankfully, their diagnostics for paternity attribution have been based on DNA profiles without mismatching. Saumitou‐Laprade et al. (2017) have not reported any mismatching, which is surprising, based on the method employed.

Consequently, the DSI model is probably useful to draw continuity between the SI systems in Oleaceae–*Phillyrea*–*Fraxinus*–*Olea* – this is an important opened question. However, no comment was given on the Jasmineae tribe of this family that displays architecture SI (Olesen, Dupont, Ehlers, Valido, & Hansen, 2005). Breton, Villemur, and Bervillé (2017) basing themselves on cross data obtained by Cáceres et al. (2015) between *Olea europaea* subsp. *cuspidata* and *Olea europaea* subsp *europaea*, showed that the SI system also functions in subsp. *cuspidata*. Besnard, Baali‐Cherif, Bettinelli‐Riccardi, Parietti, and Bouguedoura (2009) suggested gametophytic SI in *O. e. laperrinei*. Data are lacking on SI in other subsp. of *Olea*, thus homomorphic sporophytic DSI has not been shown in the whole *Olea europaea* L. species, but only in the subsp. *europaea* var. *europaea* (the cultivated form), and not in the wild olive tree (var. *sylvestris*).

It is premature for Saumitou‐Laprade et al. (2017), basing themselves on the DSI model, to claim that pollination can be improved in orchard management. Indeed, difficulties appeared when fruit setting was asymmetric, which caused concerns on fruit yield. In fact, in plenty of situations, when the host variety receives compatible pollen from the pollen donor – usually it is then named the pollinizer – the pollinizer may stay nonpollinated. The most common situation is ♀Lucques [R2R3], male sterile) x ♂Cayon (R1), the cross is compatible. Then, in the other direction ♀Cayon [R1R4] x ♂Lucques (male sterile), Cayon remains without fruit, unless in the vicinity other varieties may pollinate Cayon. However, when Tanche, which shares the same S‐allele pair with Lucques and Dolce Agogia (Oit 15 in Saumitou‐Laprade et al., 2017) is the pollen donor, Cayon is sufficiently pollinated by Tanche, as ♀Cayon [R1R4] x ♂Tanche (R2) is compatible. Tanche is partially male sterile (Besnard et al., 2000). Symmetric crosses occur when the two varieties do not share the same S‐allele pair, and when both S‐determinants are present on the pollen coat.

♀Tanche x ♂Salonenque and ♀Salonenque x ♂Tanche are incompatible in both directions. So, the *R5* S‐allele was attributed to Salonenque [R3R5] and to Grossane. Indeed, Grossane cannot cross with Salonenque, but can cross with Tanche: ♀Tanche [R2R3] x ♂Grossane (R1R5) succeeded, whereas ♀Salonenque [R3R5] x ♂Grossane (R1R5) failed, thus shot berries appeared (Koubouris, Metzidakis, & Vasilakakis, 2010). Grossane and Leccino (Oit 27 in Saumitou‐Laprade et al., 2017) both harbor *R1R5* and because of co‐dominance of *R1* and *R5* (*R1 *=* R5*) the pollen is coded R1R5. Aglandau is one of the pollinizers of Grossane, ♀Grossane [R1R5] x ♂Aglandau (R2), whereas in the other direction pollen from Grossane is incompatible with Aglandau, ♀Aglandau [R3R5] x ♂Grossane (R1R5) failed.

Moreover, Saumitou‐Laprade et al. (2017) have explained only half of crosses between olive varieties, and claimed that the host variety had 50% chance to match compatibility with the pollen donor. This assertion is based on prediction from DSI and remains to be experimented in orchards. Farinelli et al., 2015, (see the Table 4) have shown that each host variety has a different probability to match with a pollen donor, that renders complex future orchard composition to equilibrate host varieties and pollinizers. For those orchards, already in production, the recommendation to enhance pollination is to introduce new pollen sources by grafting or planting different pollinizers to ensure correct pollination.

## 2 CONCLUSION

Our goal was to improve the clarity of SI in the olive – here olive means the cultivated form. Olive growers will probably not be interested by these exchanges unless they can identify the olive materials. To summarize, the pertinent points addressed in the letter, at least for us are,

1. 1:1 segregation of SI should be checked in two different pseudo‐backcross offsprings.
2. The main progenies should be identified (which denomination is Oit64?) as the materials given in tables to enable comparison with published data.
3. The work described in Saumitou‐Laprade et al. (2017) is a verification of the DSI model, and it is the first step. The second step is to predict, for chosen pairs of varieties after crosses in both directions, that fruit set is symmetric (they will succeed or they will fail in both directions), and the third step is to predict for some other crosses – in both directions – that they succeed in one direction and fail in the other direction. Then, we would see comparison of prediction based on pollen germination and experimental data for fruit setting. We have gone in Breton et al. (2014) and Farinelli et al. (2015) through these steps successfully for more than 100 pairwise combinations of varieties.
4. Fruit numbers were not referred to hermaphroditic flowers, thus the fruit number under a bag has no meaning when comparing fruit setting between varieties. This point is the key problem in most olive studies.
5. Marchese et al. (2016) have eliminated most profiles (supposed to correspond to one embryo) because they have more than two SSR alleles at some loci. Consequently, using seedlings helps to avoid the problem to mix embryos, but delays the data for 1 year. The most probable father has never been verified by other independent method(s), and nobody has published the verification of the compatibility between the putative father and the host based on a controlled cross.

Finally, Saumitou‐Laprade et al. (2017) found differences between pollen germination tests (data are qualitative, all or nothing) leading to all their inferences, and the bag method, which provided fruit set quantitative data leading to other inferences (Table 1). This is sustained in senecio (Brennan, Harris, Tabah, & Hiscock, 2002), chicory (Gonthier et al., 2013), in sunflower (Nooryazdan, Serieys, David, Baciliéri, & Bervillé, 2010), and here for the olive. Consequently, inferences from pollination germination tests remain to be conciliated with those from fruit set data under bags.

### DATA ARCHIVING

Raw data are published in quoted articles from Breton et al. (2014), Farinelli et al. (2015) Koubouris et al. (2014), Breton et al. (2016, 2017).
